# Protective Effects of Nootkatone on Renal Inflammation, Apoptosis, and Fibrosis in a Unilateral Ureteral Obstructive Mouse Model

**DOI:** 10.3390/nu13113921

**Published:** 2021-11-01

**Authors:** Chang-Mu Chen, Chen-Yu Lin, Yao-Pang Chung, Chia-Hung Liu, Kuo-Tong Huang, Siao-Syun Guan, Cheng-Tien Wu, Shing-Hwa Liu

**Affiliations:** 1 Division of Neurosurgery, Department of Surgery, College of Medicine and Hospital, National Taiwan University, Taipei 10051, Taiwan; cmchen10@ntuh.gov.tw; 2 Institute of Toxicology, College of Medicine, National Taiwan University, Taipei 10051, Taiwan; d06447002@ntu.edu.tw (C.-Y.L.); bnbn0506@gmail.com (Y.-P.C.); 3 Department of Urology, School of Medicine, College of Medicine, Taipei Medical University, Taipei 11041, Taiwan; mexxliu@tmu.edu.tw; 4 TMU Research Center of Urology and Kidney, Taipei Medical University, Taipei 11041, Taiwan; 5 Department of Urology, Shuang Ho Hospital, Taipei Medical University, New Taipei City 23561, Taiwan; 6 Department of Nephrology, Department of Internal Medicine, National Taiwan University Hospital, Taipei 10051, Taiwan; d97447003@ntu.edu.tw; 7 Institute of Nuclear Energy Research, Atomic Energy Council, Taoyuan 32546, Taiwan; ssguan@iner.gov.tw; 8 Department of Nutrition, China Medical University, Taichung 406040, Taiwan; 9 Master Program of Food and Drug Safety, China Medical University, Taichung 406040, Taiwan; 10 Department of Medical Research, China Medical University Hospital, China Medical University, Taichung 406040, Taiwan; 11 Department of Paediatrics, National Taiwan University Hospital, Taipei 10051, Taiwan

**Keywords:** chronic kidney disease, nootkatone, inflammation, apoptosis, renal fibrosis

## Abstract

Nootkatone is one of the major active ingredients of *Alpiniae oxyphyllae*, which has been used as both food and medicinal plants for the treatment of diarrhea, ulceration, and enuresis. In this study, we aimed to investigate whether nootkatone treatment ameliorated the progression of chronic kidney diseases (CKD) and clarified its underlying mechanisms in an obstructive nephropathy (unilateral ureteral obstructive; UUO) mouse model. Our results revealed that nootkatone treatment preventively decreased the pathological changes and significantly mitigated the collagen deposition as well as the protein expression of fibrotic markers. Nootkatone could also alleviate oxidative stress-induced injury, inflammatory cell infiltration, and renal cell apoptotic death in the kidneys of UUO mice. These results demonstrated for the first time that nootkatone protected against the progression of CKD in a UUO mouse model. It may serve as a potential therapeutic candidate for CKD intervention.

## 1. Introduction

Chronic kidney disease (CKD) is one of the major public health problems worldwide with a global prevalence of 9.1% (approximately 697.5 million cases), causing 1.2 million annual deaths. According to the statistical data between 1997 and 2017, the prevalence saw an increase of 29.3%, while the incidence of dialysis and kidney transplantation was also elevated by 32.8% and 21.6%, respectively [1]. These estimates suggest an urgent need for the improvement of CKD and the importance of finding targeted treatment. The development of kidney fibrosis is one of the important characteristics of biological and pathological changes in CKD subjects. Certain fibrotic molecules, such as the fibroblast growth factor (FGF), the connective tissue growth factor (CTGF), and the transforming growth factor (TGF)-β, contribute to extracellular matrix (ECM) accumulation and the induction of type II epithelial–mesenchymal transition (EMT) [2]. The fibroblasts can also proliferate and express α-smooth muscle actin (α-SMA), which is identified as a renal fibrotic marker [3]. In addition, during the progressive tubulointerstitial nephropathy, the damaged or apoptotic cells attract the infiltration of inflammatory cells, such as macrophages [4] and granulocyte [5], to induce inflammatory responses, leading to the progression of renal injury. Therefore, anti-fibrosis has recently been considered to be a novel strategy for treating CKD [6].

*Alpiniae oxyphyllae* has been classified as an edible fruit and healthy food. It has also been broadly used as the traditional medicine for the treatment of diarrhea, ulceration, and enuresis [7]. Its fruit extracts revealed an anti-hyperuricemic effect in the kidney [8], hepatoprotective function against CCl_4_-induced oxidative damage in vitro and in vivo [9], and anti-diabetic action [10]. Recent pharmacological evidence indicated that one of the important active ingredients of *A. oxyphyllae*, nootkatone (NKT), exhibited excellent protective and inhibitory effects on inflammation and apoptosis in neuronal cells [11], displayed improved properties against particle-induced lung injury in mice, and also prevented fibrotic actions and inflammatory response in a liver injury murine model [12]. These empirical and intensive studies suggested potential benefits and applications of NKT in the improvement of diseases. However, whether the administration of NKT alleviates the progression of CKD remains uncertain.

In this study, we investigated the potential therapeutic efficacy and underlying mechanisms of oral NKT treatment in a mouse model with unilateral ureteral obstruction (UUO)-induced renal fibrosis. The experimental UUO mouse model is a stable and well-established procedure to elicit oxidative stress and inflammatory response, apoptotic cell death, extracellular matrix (ECM) accumulation, and fibrotic development in the kidney [13].

## 2. Materials and Methods

### 2.1. Animal Care, NKT Treatment, and UUO Surgical Procedure

Male C57BL/6J mice, 6–8 weeks old, were purchased from the laboratory animal center of the College of Medicine, National Taiwan University. The protocol of this animal study was approved by the Laboratory Animal Care and Welfare Committee, College of Medicine, National Taiwan University. All mice were humanely cared for and housed in a specific pathogen-free room with food and water ad libitum. The temperature was maintained at 22 ± 2 °C with a 12 h light–dark cycle. After a week of acclimation, mice were randomly divided into sham, UUO, NKT treatment (10 mg/kg/day; Sigma-Aldrich, St. Louis, MO, USA; purity ≥ 98%), and NKT treatment + UUO groups for 14 or 28 days (*n* = 6). UUO surgery was a progressive CKD mouse model as described previously [14]. For the UUO surgical procedure, mice were anesthetized with 3% isoflurane and an abdominal incision was made around the left kidney, then the ureter was ligated by a cotton thread under the heating plate (36 °C). NKT was freshly prepared with sterile water to the final working solution (10 mg/kg with the volume of 10 mL/kg), and orally administrated to mice for 14 or 28 consecutive days. Mice were subsequently euthanized under anesthesia for the experimental detections that followed. Candesartan (Sigma-Aldrich, St. Louis, MO, USA; 5 mg/kg/day) was used as a positive control as described previously [15].

### 2.2. Histopathological Detection

The isolated kidneys from sham, UUO, and treatment groups were immersed in the 4% formalin/phosphate buffer solution (PBS), and samples were fixed for one week. After paraffin embedding, the 4 μm-thick sections were stained with Hematoxylin and Eosin (H&E) to detect the histopathological changes, and then the injury levels were scored as described previously [16]. Briefly, sections were deparaffinized and dehydrated with 70%, 85%, and 95% ethanol/PBS buffer, and then the nucleus and cytoplasm were stained with the H&E staining buffer (Sigma-Aldrich, St. Louis, MO, USA). Renal injury scoring was assessed with tubular dilation, necrotic tubular cells, glomerulosclerosis, interstitial infiltration, or proliferative fibroblast cells through 15 random fields of renal cortical areas. Abnormal areas were examined using blind evaluation with four-point scores of 0 = normal, 1 = mild (<25%, abnormal pathology injury), 2 = moderate (25–50%), 3 = severe (50–75%), and 4 = large area injury (>75%). For Mason’s trichrome staining, 4 μm-thick renal sections were prepared to assess the collagen deposition as described previously [17]. Briefly, kidney sections were deparaffinized, rehydrated, and stained in Bouin’s fluid, Weigert’s iron hematoxylin working solution, and the aniline blue solution according to the manufacturer’s protocol guide (Sigma-Aldrich, St. Louis, MO, USA). The positive staining section areas were identified as the collagen deposition, which was quantified by Fovea Pro 4.0 imaging software (Reindeer Graphics, Asheville, NC, USA).

### 2.3. Western Blotting

The detection of protein expression was performed by Western blotting as described previously [18]. Briefly, kidney samples were collected and lysed in the RIPA buffer (Millipore Technology, Billerica, MA, USA). After boiling, 20 μg samples were loaded into the sodium dodecyl sulfate-polyacrylamide gel electrophoresis (SDS-PAGE) (8–12%), transferred to the PVDF membrane (Millipore Technology, Billerica, MA, USA), and then blocked with 5% bovine serum albumin (BSA). Subsequently, the PVDF membrane was incubated with primary antibodies overnight at 4 °C, including fibronectin (BD Biosciences, San Jose, CA, USA), α-SMA (Sigma-Aldrich, St. Louis, MO, USA), E-cadherin, phosphorylated NFκB-p65 (p-p65), the total form of NFκB-p65, cleaved PARP, cleaved caspase 3, α-tubulin, cyclooxygenase-2 (COX-2), interleukin-1β (IL-1β), and CTGF (Santa Cruz Biotechnology, Santa Cruz, CA, USA), and then incubated in the secondary antibody (Santa Cruz Biotechnology, Santa Cruz, CA, USA). Finally, the protein expression levels of the PVDF membrane were detected by the enhanced chemiluminescence kit (Millipore Technology, Billerica, MA, USA) in a digital photo-image system.

### 2.4. Fluorescent Terminal Deoxynucleotidyl Transferase dUTP Nick End Labeling (TUNEL) Staining

The 4 μm-thick formalin-fixed and paraffin-embedded renal sections were used to assess the apoptotic cells by immunofluorescence TUNEL staining as described previously [17]. Briefly, kidney sections were deparaffinized and rehydrated with 50–90% ethanol/PBS buffer. Subsequently, the protein was digested by proteinase K for 20 min, washed with PBS buffer, and then stained with a TUNEL assay kit (Promega, Madison, WI, USA). Samples were finally counter-stained by the Hoechst 33,258 (Sigma-Aldrich, St. Louis, MO, USA) and the slides were mounted to detect TUNEL-positive staining cells under fluorescence microscopy with 200× magnification. The 15 random fields were examined using a blind evaluation to choose and count.

### 2.5. Immunohistochemical (IHC) Staining

The 4 μm-thick formalin-fixed and paraffin-embedded renal sections were used to detect the inflammatory responses by IHC staining as described previously [15]. Briefly, sections were deparaffinized and dehydrated with 50–90% ethanol/PBS buffer, immersed in the citrate buffer solution (pH 6.0), and then boiled for antigen retrieval. After blocking with 5% BSA, sections were incubated in a 3% hydrogen peroxide/methanol solution to deplete the peroxidase, and then reacted with primary antibodies for LY6G (eBioscience, San Diego, CA, USA) and F4/80 (Cell Signal Technology, Danvers, MA, USA). Finally, sections were color-displayed with the Polymer-HRP linker and a 3,3′-diaminobenzidine tetrahydrochloride detection kit (BioGenex, Fremont, CA, USA). The positively stained areas were identified and quantified by Fovea Pro 4.0 imaging software (Reindeer Graphics, Asheville, NC, USA).

### 2.6. Statistical Analysis

All results were collected and presented as mean ± S.D. The *p*-value < 0.05 was considered a significant difference between the sham control and treatment groups, which was calculated by one-way analysis of variance (ANOVA) followed by a post hoc analysis with Tukey HSD. SigmaPlot (version 12.0; Systat Software, San Jose, CA, USA) software was used in this study.

## 3. Results

### 3.1. NKT Treatment Ameliorates Renal Fibrosis and Pathological Changes in the UUO Kidneys

**Figure 1:**
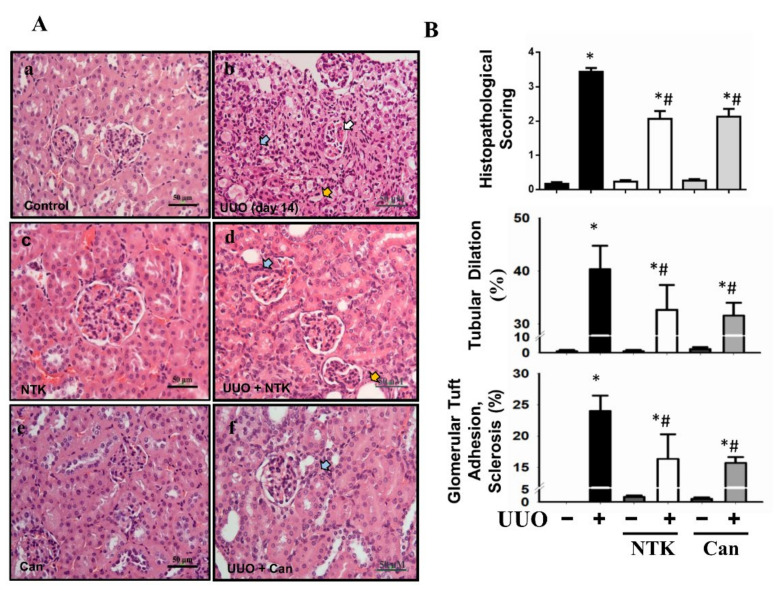
Effects of Nootkatone treatment on the renal pathological changes in a UUO mouse model. Vehicle, Nootkatone (NKT), or candesartan (Can; positive control) was orally given to the sham or UUO mice for 14 days. (**A**) The pathological changes were determined by hematoxylin and eosin (H&E) staining. The kidney injury features, including kidney tubular dilation (yellow arrow), interstitial infiltration (blue arrow), and glomerular tuft adhesion and sclerosis (white arrow) were indicated. Scale bar: 50 μm. (**B**) The histopathological scoring is shown Data are presented as mean ± SD (*n* = 6). * *p* < 0.05 vs. the sham control group. # *p* < 0.05 vs. the UUO group.

**Figure 2:**
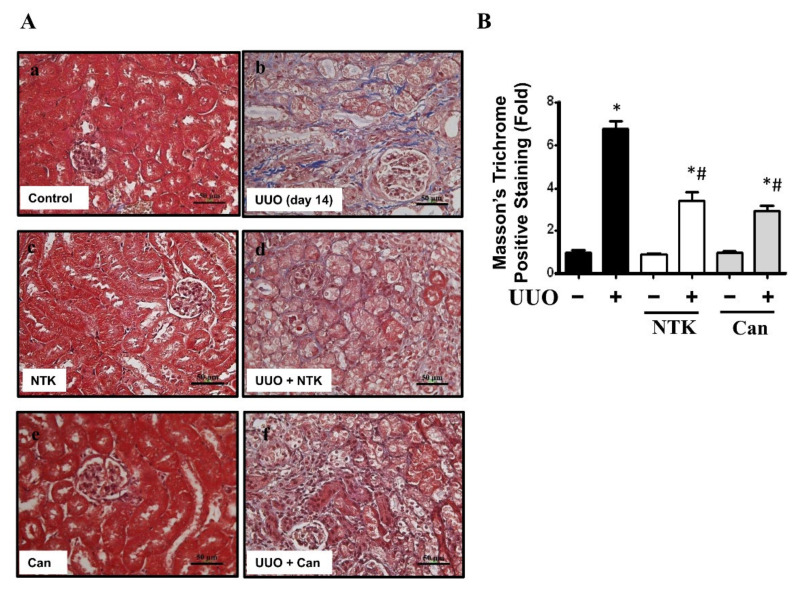
Effects of Nootkatone treatment on the collagen deposition in the kidneys of UUO mice. Vehicle, Nootkatone (NKT), or candesartan (Can; positive control) was orally given to the sham or UUO mice for 14 days. Collagen deposition was determined by Masson’s Trichrome staining (**A**). a, Control; b, UUO; c, NTK; d, UUO+NTK; e, Can; f, UUO+Can. Scale bar: 50 μm. The quantification is shown in (**B**). Data are presented as mean ± SD (*n* = 6). * *p* < 0.05 vs. the sham control group. # *p* < 0.05 vs. the UUO group.

**Figure 3:**
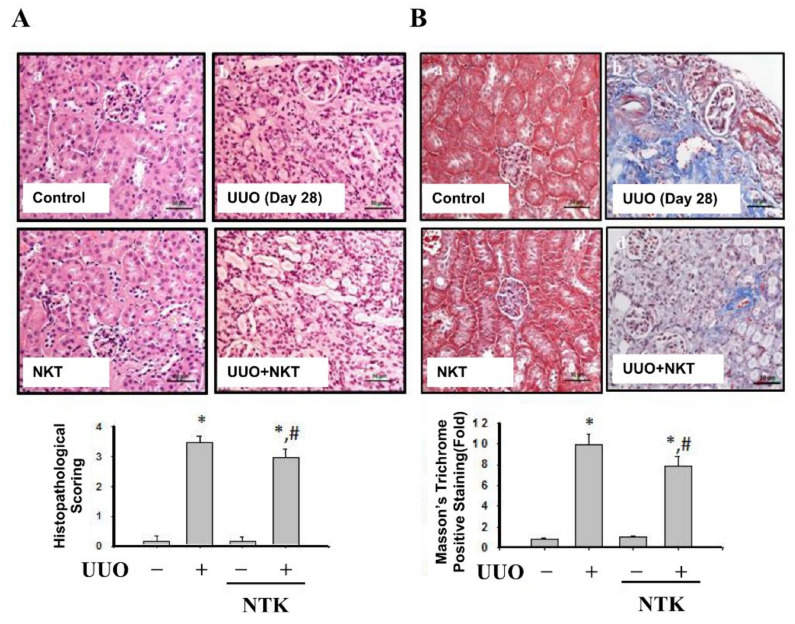
Effects of Nootkatone treatment on the renal pathological changes and collagen deposition in a UUO mouse model. Vehicle or Nootkatone (NKT) was orally given to the sham or UUO mice for 28 days. The pathological changes determined by H&E staining (**A**). The collagen deposition was determined by Masson’s trichrome staining (**B**). Scale bar: 50 μm. Data are presented as mean ± SD (*n* = 6). * *p* < 0.05 vs. the sham control group. # *p* < 0.05 vs. the UUO group.

Renal fibrosis is an important feature of CKD [6]. To investigate whether NKT treatment retarded the progression of CKD, we first observed the histopathological changes in the kidneys of UUO mice. Candesartan, which is an angiotensin type II receptor blockade and has alleviated interstitial fibrosis in UUO mice [19], was used as a positive control [20]. As shown in Figure 1, there were obvious pathological changes in the kidneys of UUO mice over 14 days with kidney injury features, including tubular dilation, interstitial infiltration, and glomerular tuft adhesion and sclerosis. Treatment with both NKT and Candesartan for 14 days significantly reversed the pathological changes in the kidneys of UUO mice (Figure 1). Obvious collagen disposition was observed in the renal tubular and interstitial areas of UUO mice, which could be significantly reversed by both NKT and Candesartan treatment (Figure 2). Furthermore, NKT treatment also prevented histopathological changes (Figure 3A) and collagen deposition (Figure 3B) in the kidneys of UUO mice for 28 days. However, the effects of NKT on UUO kidneys at 28 days were not better than the effects of NKT on UUO kidneys at 14 days.

**Figure 4:**
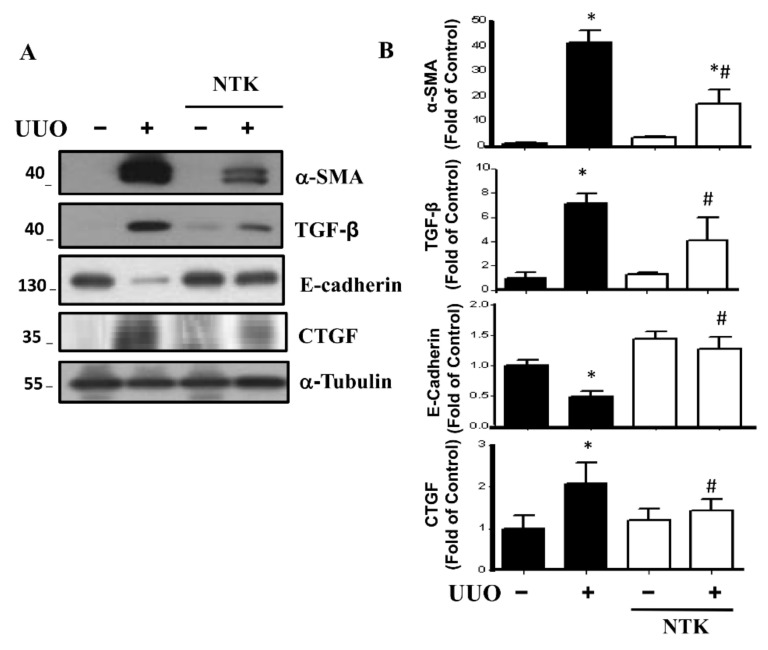
Effects of Nootkatone treatment on the renal fibrosis in the UUO kidneys. Vehicle or Nootkatone (NKT) was orally given to the sham or UUO mice for 14 days. Protein expression levels of the fibrotic signaling markers were determined by Western blotting (**A**). The quantification is shown in (**B**). Data are presented as mean ± SD (*n* = 6). * *p* < 0.05 vs. the sham control group. # *p* < 0.05 vs. the UUO group.

We next determined the protein expression levels of the fibrotic markers α-SMA, TGF-β, and CTGF, as well as an EMT marker E-cadherin [21,22,23]. As shown in Figure 4, the fibrotic markers were significantly upregulated, while E-cadherin was downregulated in the kidneys of UUO mice. Intriguingly, after NKT treatment for 14 days, the increased protein expression levels of fibrotic markers and the decreased protein expression of E-cadherin were significantly mitigated in the UUO kidneys. These results suggest that oral administration of NKT prevents the progression of renal fibrosis in the kidneys of UUO mice.

### 3.2. Administration of NKT Mitigates Oxidative Stress Injury and Inflammatory Cell Infiltration in the UUO Kidneys 

**Figure 5:**
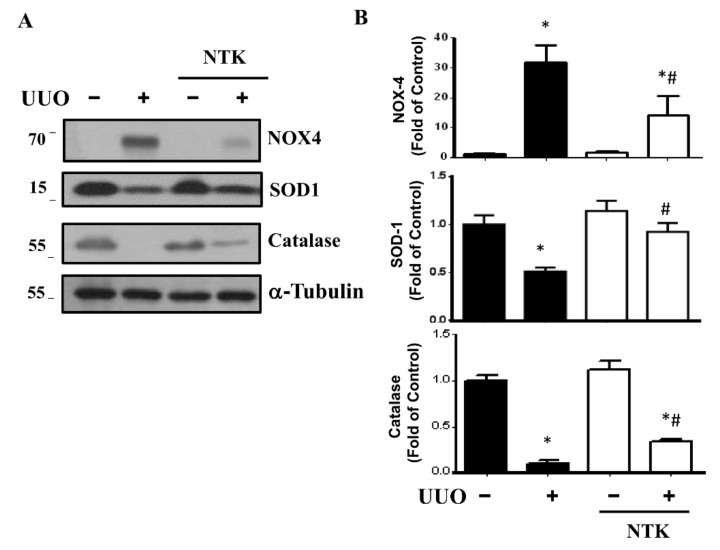
Effects of Nootkatone treatment on oxidative stress injury in the UUO kidneys. Vehicle or Nootkatone (NKT) was orally given to the sham or UUO mice for 14 days. Protein expression levels of the oxidative stress markers were determined by Western blotting (**A**). The quantification is shown in (**B**). Data are presented as mean ± SD (*n* = 6). * *p* < 0.05 vs. the sham control group. # *p* < 0.05 vs. the UUO group.

**Figure 6:**
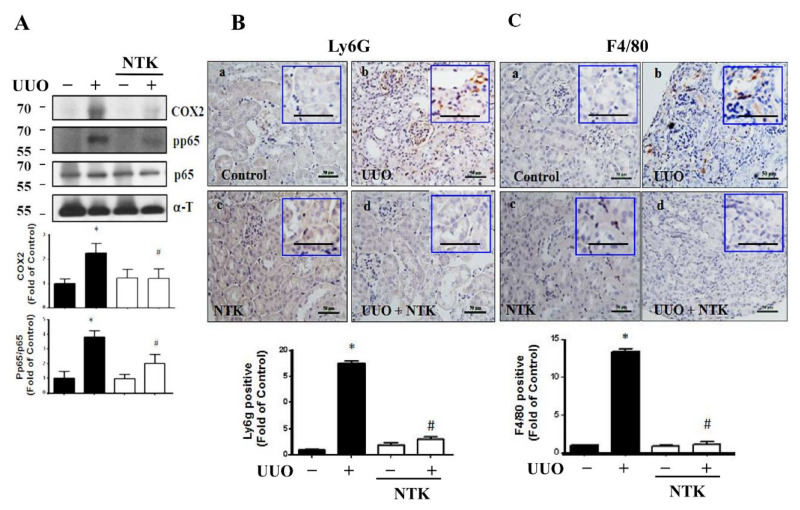
Effects of Nootkatone on the inflammatory cell infiltration in the UUO kidneys. Vehicle or Nootkatone (NKT) was orally given to the sham or UUO mice for 14 days. Protein expression levels of pro-inflammatory signaling molecules were determined by Western blotting (**A**). The changes in the protein expression of both neutrophil marker Ly6G and macrophage marker F4/80 by IHC are shown in (**B**,**C**), respectively. a, Control; b, UUO; c, NTK; d, UUO+NTK. Scale bar: 50 μm. Data are presented as mean ± SD (*n* = 6). * *p* < 0.05 vs. the sham control group. # *p* < 0.05 vs. the UUO group. α-T: α-Tubulin.

Oxidative stress plays an important role in the fibrosis and progression of CKD [24]. The persistent induction of reactive oxygen species (ROS) would arouse inflammatory responses including the enhancement of neutrophils or macrophage recruitment and the subsequent interstitial fibrosis as well as glomerulosclerosis in the UUO mice [25,26]. Interestingly, NKT treatment has been found to improve chemical or hyperammonemia-induced oxidative stress in liver injury rodent models [9,27], as well as attenuate myocardial oxidative damage and inflammation in a myocardial infarction rat model [28]. Therefore, we next examined the protective effects of NKT treatment on the oxidative stress and inflammatory responses in the kidneys of UUO mice. As shown in Figure 5, the levels of protein expression for antioxidant enzymes SOD1 and catalase were dramatically decreased in the kidneys of UUO mice, which could be significantly reversed by NKT treatment. Furthermore, NAD(P)H oxidase-4 (Nox4) was one of the major sources of ROS that could be a target for the reduction of tubulointerstitial fibrosis [29]. As shown in Figure 5, the increased Nox4 protein expression in the kidneys of UUO mice could be attenuated by NKT treatment. Moreover, the protein expression levels of inflammation-associated signaling molecules cyclooxygenase (COX)-2 and phosphorylated NFκB-p65 were significantly augmented in the UUO kidneys, which could be significantly eliminated by NKT treatment (Figure 6A). Similarly, the levels of protein expression of Ly6G, a marker of the infiltrated neutrophils, and F4/80, a marker of the macrophage, were significantly elevated in the UUO kidneys, which could be restored by NKT treatment (Figure 6B,C). These results suggest that the inhibitory effects of oral NKT administration on inflammatory responses and oxidative stress may contribute to an improvement in the progression of renal fibrosis in a UUO mouse model.

### 3.3. NKT Treatment Protects against Apoptotic Cell Death in the UUO Kidneys

**Figure 7:**
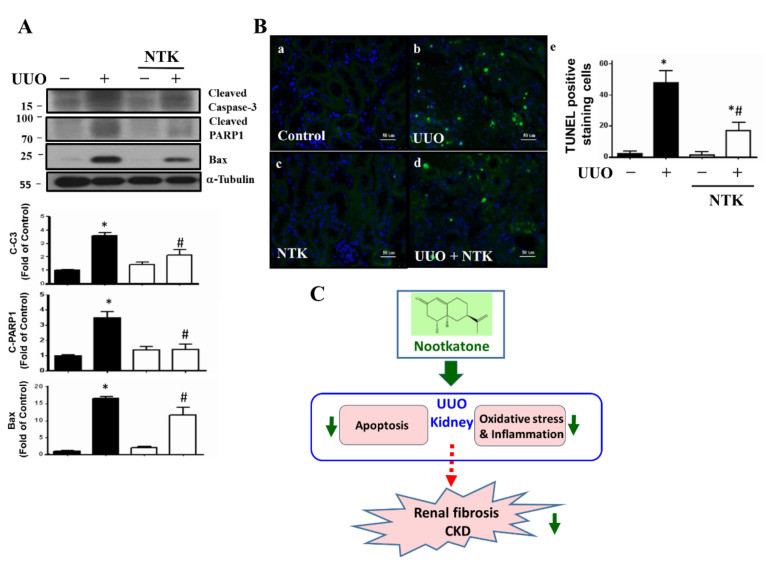
Effects of Nootkatone treatment on the renal cell apoptosis in the kidneys of UUO mice. Vehicle or Nootkatone (NKT) was orally given to the sham or UUO mice for 14 days. Protein expression levels of apoptotic molecules were determined by Western blotting (**A**). The TUNEL-positive cells are shown in (**B**). Data are presented as mean ± SD (*n* = 6). a, Control; b, UUO; c, NTK; d, UUO+NTK. * *p* < 0.05 vs. the sham control group. # *p* < 0.05 vs. the UUO group. C-C3: Cleaved Caspase-3; C-PARP1: Cleaved PARP1. (**C**) A schematic summary of our main findings for the effects of NKT on the progression of chronic CKD in a UUO mouse model.

Apoptotic cell death has been demonstrated to be a pivotal event for the progression of renal fibrosis with the regulation of Bax-associated signaling family during the process of experimental obstructive-induced nephropathy [30,31]. Additionally, NKT treatment has been found to ameliorate inflammation and apoptosis in rats with isoproterenol-induced myocardial infarction [28] and in mice with diesel-exhaust-particles-induced lung injury [32]. We next examined whether NKT treatment protected against renal cell apoptosis in the UUO mouse kidneys. As shown in Figure 7A, the levels of protein expression for cleaved PARP, cleaved Caspase-3, and Bax were significantly elevated in the UUO kidneys, which could be significantly reversed by NKT treatment. Moreover, the numbers of TUNEL-positive staining cells were significantly increased in the UUO kidneys, which could also be significantly reversed by NKT treatment (Figure 7B). These results suggested that NKT treatment could effectively protect against apoptotic cell death during the process of obstructive nephropathy.

## 4. Discussion

The UUO animal model displayed the progression of obstructive nephropathy to final renal fibrosis. Several events such as tubular dilation, epithelial cell death, loss of proximal tubular mass, infiltration of leukocytes, oxidative stress and inflammation induction, glomerulosclerosis, and interstitial fibrosis were observed [14]. This model has certain limitations and cannot fit all types of CKDs [14]. However, the UUO model still has a high-throughput property and easily typified CKD in a relatively short procedure, and can serve as a common model for testing the preventive effects of drugs on renal fibrosis [14,33]. Obstructive nephropathy is also a significant health problem in the world, which can be classified as a secondary injury of acute kidney injury (AKI). It is a frequent event that can be accounted for in around 10% of all AKI cases [34] and up to 22% in elder subject s [35]. Another report indicated that the kidney stone, an obstructive disorder in the kidney, had a 51 to 68% increased risk for CKD by diagnosis and 25% to 44% increased risk for CKD by serum creatinine level judgment [36]. A large population-based historical cohort study by Hippisley-Cox et al. found that stone-former women have a 27% increased adjusted risk for moderate to severe CKD [37]. Furthermore, Stankus et al. have observed that the prevalence of pre-end-stage renal disease (ESRD) kidney stones in African–American hemodialysis patients is significantly higher than the prevalence of kidney stones in African–American persons, obtained by the Third National Health and Nutrition Survey [38]. These empirical data suggested that obstructive nephropathy is related to the progression of CKD.

Renal fibrosis, an important feature and a hallmark correlated with progressive CKD, is commonly accepted as key to regulating the progression of CKD [39]. Menn-Josephy et al. have shown that renal interstitial fibrosis is imperfect but performed well overall as a predictor of progression to ESRD [40]. They have found that the mean interstitial fibrosis on kidney biopsy is about 31.2%, the mean glomerulosclerosis is about 25.0%, and patients with >50% interstitial fibrosis progressed more rapidly on average than patients with either 25–49 or 0–24% interstitial fibrosis to ESRD with about 1.2, 6.5, and >10 year median time, respectively [40]. Several studies have also shown that the inhibitors for interstitial fibrosis-related molecular factors protect against CKD progression [41], and tubulointerstitial fibrosis changes can be understood as a marker in the progression of renal damage [42] or renal failure [43].

Candesartan, an angiotensin II receptor blocker, has been interpreted to have anti-fibrotic benefits in a UUO mouse model [44]. Our previous study further demonstrated that candesartan could also ameliorate renal fibrosis through the inhibition of endoplasmic reticulum (ER)-stress-induced apoptosis [20]. In the UUO kidneys, candesartan was found to restore the levels of XBP-1 protein expression and suppress the levels of protein expression for ATF4, cATF6, and CHOP [20]. However, in our preliminary study, we observed that NKT treatment could not significantly change the protein expression levels of ER-stress-related signaling molecules in the UUO kidneys (data not shown). A prospective study, which compared the renoprotective effects of candesartan and the ACE inhibitor in patients with moderate renal impairment, found that candesartan possessed stronger suppression in mesangial matrix growth and interstitial fibrosis than the ACE inhibitor [45]. To identify the protective effectiveness of anti-fibrosis of nootkatone, we chose candesartan, which has been used in the clinical setting and displayed an anti-fibrotic benefit, as a positive control for the anti-fibrotic effect in the UUO model.

NKT is one of the important bioactive integrands of the medicinal plant *A. oxyphylla*. It has also been isolated from the edible fruit, *Citrus changshan-huyou* [9,46]. It is abundantly present in fruits and peels and is easily extracted by ethanol [47,48]. The huge amount of NKT and simple extraction mean it is easily obtained and has been applied as a functional ingredient of health foods or medical agents to prevent disorders or diseases. More interestingly, the extracts of *A. oxyphylla* have been used to improve urinary and renal functions, such as polyuria, hyperuricemia [8], and diabetic nephropathy in animal models [10,49]. These findings provided empirical evidence to speculate a possible excellent preventive candidate such as NKT to mitigate the progression of kidney diseases. In the present study, we demonstrated for the first time that NKT could effectively prevent renal fibrosis in a UUO mouse model. On the other hand, NKT has been shown to improve the hyperammonemia-induced oxidative stress in a D-galactosamine-induced liver injury mouse model [27], relieve cognitive impairment in a lipopolysaccharide (LPS)-induced Alzheimer’s disease mouse model [50], and attenuate myocardial cell apoptosis, oxidative damage, as well as inflammatory response in an isoproterenol-induced myocardial infarction rat model [28]. The present study also found that NKT could ameliorate the inflammatory response, oxidative stress, and apoptosis in the kidneys of UUO mice. These findings from various animal models support the idea that NKT possesses anti-inflammatory and anti-oxidative properties and could prevent cell apoptotic death in diseases or tissue injuries, such as Alzheimer’s disease [50,51], liver injury [12,27], myocardial injury [28,52], lung injury [32], and CKD (the present study).

Wang et al. demonstrated that oral administration of 5 or 10 mg/kg/day NKT contracted LPS-induced high expression of inflammatory cytokines and related signals including IL-1β, NLRP3, and NFκB in a neuroinflammatory mouse model [50]. Meeran et al. found that oral treatment with 10 mg/kg/day of NKT modulated the TLR4-NFκB signaling pathway and also mitigated the oxidative stress cascade in an isoproterenol-induced myocardial injury rodent model [2]. The protective effects of NKT 10 mg/kg/day administered for 10 consecutive days on the inhibition of acute and chronic inflammatory responses via the COX-2 signaling pathway have been revealed in a mice model [53]. Another study indicated that oral treatment of NKT 90 mg/kg improved diesel-exhaust-particles-induced lung injury via the NFκB signaling pathway in a rat exposure model. In the present study, our results also found that the administration of 10 mg/kg/day NKT for 14 days in a CKD mouse model prominently relieved oxidative stress injury, attenuated inflammatory cell infiltration, and suppressed the expression of inflammatory markers, such as NFκB and COX-2 in the kidneys. Our present results and other empirical evidence suggest the protective effects of NKT treatment against the prolonged oxidative stress as well as inflammatory responses in the progression of CKD.

Krudi et al. found that oral administration of NKT conferred anti-fibrotic and hepatoprotective actions against inflammation and apoptosis in a mouse model of liver fibrosis [12]. Xie et al. demonstrated that *A. oxyphylla* extracts possessed powerful antioxidant activity and improved diabetic nephropathy in *db/db* genetic diabetic mice [49]. Another study also suggested a protective effect of *A. oxyphylla* extracts on diabetic nephropathy, which changed the expression of the microRNA (miRNA) profile in the kidneys of *db/db* diabetic mice [10]. In this study, we further elucidated that NKT, one functional ingredient of *A. oxyphylla*, alleviated the expression of renal fibrotic markers and inflammatory markers, collagen deposition, and apoptosis in the kidneys of UUO mice. These findings suggest that oral treatment of NKT reveals anti-fibrotic benefits in the progression of CKD.

In the present study, we do not know the point at which NKT first acted, which is a limitation of this study. Nevertheless, although obstructive nephropathy is a common illness worldwide, its pathophysiological changes remain uncertain and clinical practice is controversial. The molecular mechanisms of renal injury for the experimental UUO model have been illustrated to include tubular cell apoptosis, interstitial inflammation (oxidative stress overload and inflammatory leukocyte infiltration), and interstitial fibrosis, and these three key processes in renal injury provide information beyond obstruction [54,55]. In this study, therefore, we observed the contributions of NKT treatment on these key processes, leading to the prevention of nephropathy in a UUO mouse model (Figure 7C). On the other hand, a previous study has shown that the level of 8-hydroxy-2′-deoxyguanosine (8-OHdG), a marker of oxidative DNA damage, in the kidneys of streptozotocin-induced diabetic rats is significantly enhanced, which is in parallel with the mitochondrial DNA deletion [56]. The oxidative mitochondrial DNA damage in the kidney may be involved in the pathogenesis of diabetic nephropathy [56]. Therefore, the oxidative mitochondrial DNA may also be a therapeutic target of NKT in the UUO kidney. The levels of 8-OHdG and mitochondrial DNA in the UUO kidneys in the presence or absence of NKT may need further investigation in the future.

## 5. Conclusions

The present study revealed a key insight into the protective effect of NKT treatment on the progression of CKD in a UUO mouse model. NKT treatment diminished oxidative stress and the inflammatory response, relieved apoptotic cell death, and also attenuated renal fibrosis and the accumulation of ECM. These findings demonstrate, for the first time, that administration of NKT improves renal fibrosis and the progression of CKD. Currently, there are no effective cures for the progression of CKD. NKT may serve as a potential therapeutic agent for the treatment of CKD progression in the future.

## Data Availability

The data presented in this study are available from the corresponding author upon reasonable request.

## Abbreviations

α-SMA	α-smooth muscle actin
CKD	Chronic kidney disease
CTGF	Connective tissue growth factor
EMT	Epithelial-mesenchymal transition
FGF	Fibroblast growth factor
NKT	Nootkatone
TGF-β	Transforming growth factor-beta
UUO	Unilateral ureteral obstruction
